# GBDT Method Integrating Feature-Enhancement and Active-Learning Strategies—Sea Ice Thickness Inversion in Beaufort Sea

**DOI:** 10.3390/s24092836

**Published:** 2024-04-29

**Authors:** Yanling Han, Junjie Huang, Zhenling Ma, Bowen Zheng, Jing Wang, Yun Zhang

**Affiliations:** Shanghai Marine Intelligent Information and Navigation Remote Sensing Engineering Technology Research Center, Key Laboratory of Fisheries Information, Ministry of Agriculture, College of Information, Shanghai Ocean University, Shanghai 201306, China; ylhan@shou.edu.cn (Y.H.); hjunjie1212@163.com (J.H.); zbw17377349137@163.com (B.Z.); wangjing@shou.edu.cn (J.W.); y-zhang@shou.edu.cn (Y.Z.)

**Keywords:** sea ice thickness, active learning, query strategy, GBDT, feature enhancement, sentinel-1, normalization processing

## Abstract

Sea ice, as an important component of the Earth’s ecosystem, has a profound impact on global climate and human activities due to its thickness. Therefore, the inversion of sea ice thickness has important research significance. Due to environmental and equipment-related limitations, the number of samples available for remote sensing inversion is currently insufficient. At high spatial resolutions, remote sensing data contain limited information and noise interference, which seriously affect the accuracy of sea ice thickness inversion. In response to the above issues, we conducted experiments using ice draft data from the Beaufort Sea and designed an improved GBDT method that integrates feature-enhancement and active-learning strategies (IFEAL-GBDT). In this method, the incident angle and time series are used to perform spatiotemporal correction of the data, reducing both temporal and spatial impacts. Meanwhile, based on the original polarization information, effective multi-attribute features are generated to expand the information content and improve the separability of sea ice with different thicknesses. Taking into account the growth cycle and age of sea ice, attributes were added for month and seawater temperature. In addition, we studied an active learning strategy based on the maximum standard deviation to select more informative and representative samples and improve the model’s generalization ability. The improved GBDT model was used for training and prediction, offering advantages in dealing with nonlinear, high-dimensional data, and data noise problems, further expanding the effectiveness of feature-enhancement and active-learning strategies. Compared with other methods, the method proposed in this paper achieves the best inversion accuracy, with an average absolute error of 8 cm and a root mean square error of 13.7 cm for IFEAL-GBDT and a correlation coefficient of 0.912. This research proves the effectiveness of our method, which is suitable for the high-precision inversion of sea ice thickness determined using Sentinel-1 data.

## 1. Introduction

Sea ice is an important part of the marine ecosystem, and changes in sea ice thickness are an important index for measuring global climate change. As the greenhouse effect intensifies, the global climate gradually warms, resulting in a decrease in the extent and thickness of polar sea ice. Seawater absorbs more heat than ice and snow, causing it to warm up and further accelerate the melting of sea ice. When sea ice melts, or more areas remain ice-free, it accelerates global warming, creating a vicious cycle. At the same time, the existence of sea ice has a great impact on the navigation of ships and the development of the marine industry, so the assessment and prediction of sea ice thickness have important research significance.

At present, there are two kinds of sea ice monitoring methods: the measurement method and the remote sensing method. As early as 1893, Nansen drilled holes in the Fram Strait in the Arctic [1]. While there are other methods available, such as upward-looking sonars, navigation observation, electromagnetic induction, etc., they are all time-consuming and inefficient for obtaining large-scale information on sea ice thickness. The remote sensing method is an advanced method established using satellite remote sensing technology, offering the advantages of providing large-scale synchronous observations, timeliness, and affordability. Among the available methods, optical remote sensing is easily limited by cloud cover (leading to the misidentification of sea ice) and darkness at night, and the duration of daylight hours in the polar region is short [2]. Radiation from passive microwave remote sensing can penetrate clouds and fog and is not affected by weather. Junhwa et al. used passive microwave data from AMSR2 to invert Arctic sea ice thickness, but the sensor resolution was very low [3,4]. Altimetry satellites are more suitable for scanning thick ice [5] than thin ice due to the inversion mechanisms and resolution limitations. There are some limitations in these satellite retrievals with respect to sea ice thickness.

Synthetic Aperture Radar (SAR) not only possesses the advantage of being unaffected by weather but also has a higher resolution and a coverage range of up to hundreds of kilometers, offering unique advantages in remote sensing applications [6]. Karvonen analyzed the direct correlation between the SAR backscattering coefficient and sea ice thickness in the Baltic Sea [7]. Sanden et al. used Radarsat-2 and river ice data to analyze the influence of ice thickness on the C-band and the L-band [8]. Nakamura et al. used polarimetric and interferometric (Pi) data to analyze the correlation between the polarization ratio and sea ice thickness [9,10], and Kim et al. calculated the theoretical value of the sea ice SAR depolarization effect based on the extended Bragg model [11]. Shih et al. used time series SAR images and artificially controlled indoor sea ice growth, combined with an electromagnetic scattering model and a thermodynamic growth model [12], to conduct sea ice thickness inversion. The above research was based on the empirical relationship between SAR data and sea ice thickness and has yielded good results. However, sea ice thickness inversion is usually a nonlinear problem. When the ice is thicker, not only is the linear relationship weaker, but the region also contains more sea ice types, and the effects of sea ice surface roughness, a complex dielectric constant, temperature, and salinity are more complex [13]. Therefore, the limitations of using an empirical model and single feature inversion are obvious, failing to meet the needs of high-precision thickness inversion.

Based on the above studies, in order to make full use of the features of sea ice remote sensing images and further improve the accuracy of sea ice thickness inversion, in this paper, we propose a method of integrating active-learning and feature-enhancement strategies for sample selection and feature enhancement, combined with a Gradient Boosting Decision Tree (GBDT) for sea ice thickness inversion, namely Integrated Feature-Enhancement and Active-Learning Strategies for GBDT(IFEAL-GBDT). Firstly, SAR image preprocessing and incidence angle normalization were performed to obtain normalized HH and HV characteristics. Then, the enhanced features, such as the polarization ratio and normalized difference, which are more sensitive to sea ice thickness changes, were obtained via formula calculation. Considering the influence of incidence angle on SAR data and the influence of the environment on sea ice thickness, the properties of incidence angle, sea water temperature, and time (month) were added to construct different feature vector combinations to enhance the separability of different sea ice thicknesses. In addition, the active-learning strategy based on the maximum standard deviation was studied. We took advantage of the following characteristic: the greater the information entropy, the higher the uncertainty of the samples, and the more representative samples with more information that will be selected. This affordance solves the problem wherein it is difficult to improve the accuracy of sea ice thickness inversion when the sample size is small and there is anomaly interference. Finally, combined with the GBDT method’s strong fitting ability for high-dimensional data and problems with complex feature relationships, each decision tree was used to select the best features for segmentation, more valuable sea ice feature information was mined from different feature combinations, and sea ice thickness was inverted by integrating the results of multiple decision trees. Compared with other typical sea ice thickness inversion methods, the IFEAL-GBDT method proposed in this paper achieves higher inversion accuracy.

The rest of this article is organized as follows. Section 2 details the study area and the data used. Section 3 describes the various parts of the design framework and related algorithms. Section 4 describes the relevant experimental settings and discusses and analyzes the experimental results and model parameters. Finally, the fifth section summarizes the work presented in this paper.

## 2. Research Area and Data

### 2.1. Research Area

**Figure 1:**
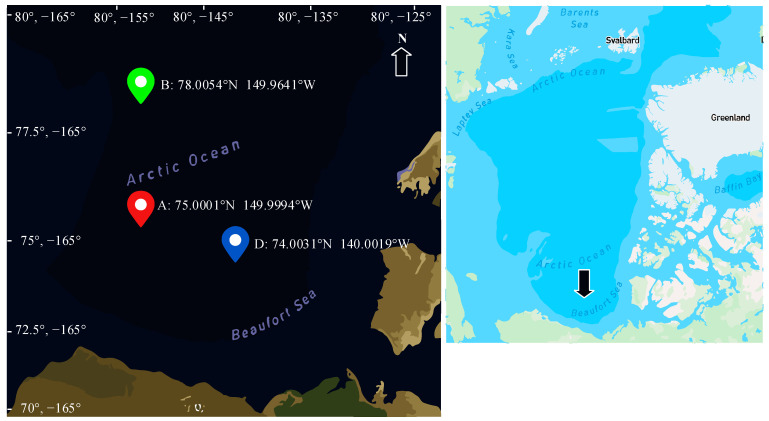
Schematic diagram of the location of the study area.

The study area is located in the Beaufort Sea in the Arctic Ocean, west of McClure Sound and north of Alaska. The study area is shown in Figure 1, where red, green, and blue dots represent upward-looking sonar (ULS) points A, B, and D, respectively. The top right corner of Figure 1 provides a small map, with a black arrow indicating the location of the Beaufort Sea in the Arctic. The area covers latitude 70° north to 80° north and longitude 160° west to 125° west, and the study period spanned from 14 September 2018, to 7 September 2021. The Beaufort Gyre Observing System (BGOS) has provided means for the continuous monitoring of the region since the summer of 2003, providing us with time series regarding temperature, salinity, ice draft, etc. [14].

### 2.2. Measured Data

Woods Hole Oceanographic Institution emitted sound waves from three sonar devices located 50 to 85 m below the BGOS ice sheet (depending on the actual mooring length and deployment depth); the vertical distance between the sonar equipment and the ice bottom was calculated based on the sound speed. Ice draft data were obtained by subtracting this vertical distance from the water depth value of the sonar equipment [14]. The sampling frequency of the sonar equipment was 2 s, so there were 43,200 data measured per day. Based on these measured data, the median, mean, maximum, minimum, and standard deviation of an ice draft could be calculated for each day. Specific information regarding the three sonar devices is shown in Table 1. Point A is about 340 km from point B, point A is about 320 km from point D, and points B and D are about 520 km away from each other. The ice thickness data can be downloaded by accessing the following link: http://www.whoi.edu/beaufortgyre (accessed on 30 September 2022).

**Figure 2:**
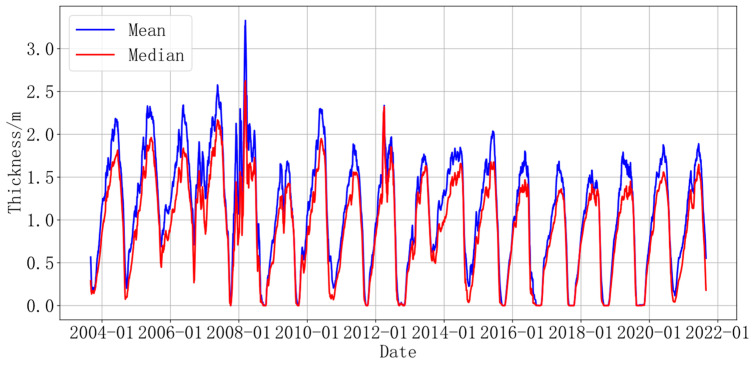
The trend of the average and median changes in ice thickness at point A (as assessed using sonar).

Figure 2 shows the daily ice thickness trend at sonar point A from August 2003 to August 2021, with the blue and red lines representing the average and median of 15 million daily data pieces, respectively. In order to smooth out errors caused by lines and measurements of sonar equipment, these values were processed using a median filter to more clearly display the data. In Equation (1),

(1) $$D_{t}=\frac{1}{20}\sum_{t-9}^{t+10}d_{t}$$

where *t* represents the day number, *d_t_* represents the thickness of sea ice on the *t*-th day, and *D_t_* represents the smoothed thickness value of sea ice on the *t*-th day, a value obtained by averaging the thickness values from the *t* − 9th day to the *t* + 10th day. From August 2003 to August 2021, the mean and median changes were cyclical. The maximum value of the mean is more than 200 cm, and the lowest is 0 cm. When the ice thickness was at its maximum during this phase, the zigzag of the lines varied greatly, and the maximum average value of each season after 2013 was lower than the pre-2013 value. More importantly, the red lines zigzag less than the blue lines, and the median is more stable, during both ice growth and ice melting.

**Figure 3:**
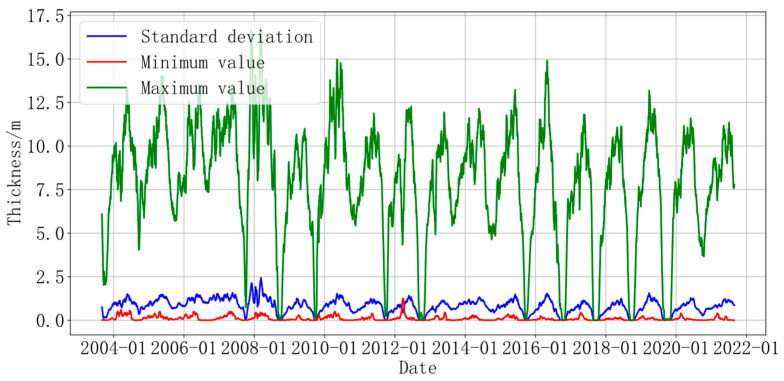
The variation trend of the standard deviation and the minimum and maximum values of ice thickness at point A (determined using sonar).

Figure 3 shows the daily ice thickness change for sonar device A from August 2003 to August 2021. The data were also processed with a median moving average, with the maximum, minimum, and standard deviation values represented by green, red, and blue lines, respectively. The maximum daily ice thickness is more than 400 cm, and the interval between maximum values during each phase is about 1 year. The daily minimum thickness is no more than 40 cm, and the trend did not change significantly after 2013, being basically close to 0 cm. The difference between the maximum and minimum daily thickness values is very large, reaching 16.7 m in March 2008, but the difference between 2007–2009, 2012, 2015, 2017, 2018, and 2019 is very small and is close to 0 cm in August–October. The standard deviation indicates the dispersion degree of the sea ice thickness change. The standard deviation fluctuates around 1 m from August to October, and the dispersion degree is greater in other months.

The accurate calculation of thickness using ice draft requires knowledge of ice type, ice density, and snow load, which introduce additional uncertainty. The ratio of ice draft to ice thickness is about 0.9 [15], and we can use the characteristics and distribution of ice draft to evaluate the satellite-derived thickness distribution. The ice thickness studied herein is thin and the effect is small, so the ice draft was not converted to the ice thickness, which can be considered to be the actual thickness value. In summary, we used the median daily data of the ice draft of the sonar devices A, B, and D to carry out relevant experiments.

### 2.3. Sentinel-1 Data

Sentinel-1 is a C-band SAR satellite launched by the European Space Agency. We used Sentinel-1’s level-1 Ground Range Detected (GRD) dual-polarization (HH and HV) products with medium spatial resolution (90 m) and up to 400 km wide coverage. By superimposing image data at different times to form time series data, we could analyze the spatio-temporal changes in and trends of sea ice. Between 14 September 2018, and 7 September 2021, there were 1854 Sentinel-1 images covering three points A, B, and D of the sonar equipment. The Sentinel-1 data can be downloaded by accessing the following link: https://search.asf.alaska.edu/#/ (accessed on 30 September 2022).

## 3. Proposed Method

### 3.1. Data Preprocessing

Before extracting Sentinel-1 image information, it is necessary to preprocess according to radar imaging characteristics in order to eliminate errors caused by system limitations. SAR data preprocessing mainly involves track correction, thermal noise removal, radiometric calibration, terrain correction, and decibelization [16]. Then, the arithmetic average is used for spatial filtering, the window size is set to 3 × 3, and finally the incidence angle is normalized.

**Figure 4:**
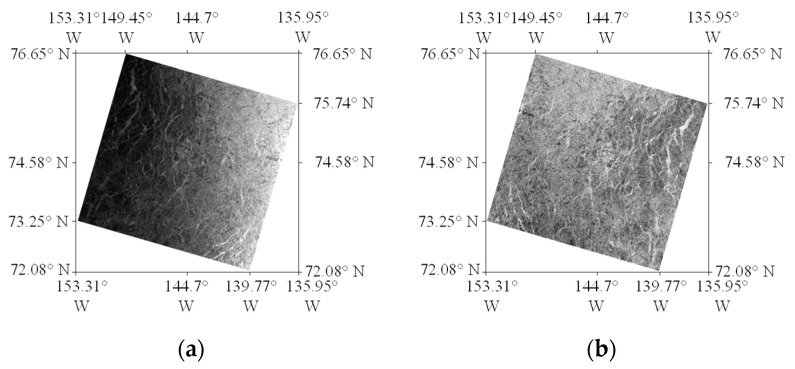
SAR image covering point A of the sonar device on 14 January 2021. (**a**) Before preprocessing. (**b**) After preprocessing.

As shown in Figure 4, the SAR image covering sonar equipment A (14 January 2021) was processed. Figure 4a shows that before preprocessing, the brightness of the image decreases significantly from right to left [16,17]. The backscattering trend of signals received in EW mode is weakened in the distance direction, seriously affecting the ability to invert sea ice thickness from wide SAR images. Thus, the correction of angle of incidence is particularly important. Figure 4b shows that, after pretreatment, the brightness of the region with a large incidence angle was significantly improved, and the overall brightness was less affected by the incidence angle.

**Figure 5:**
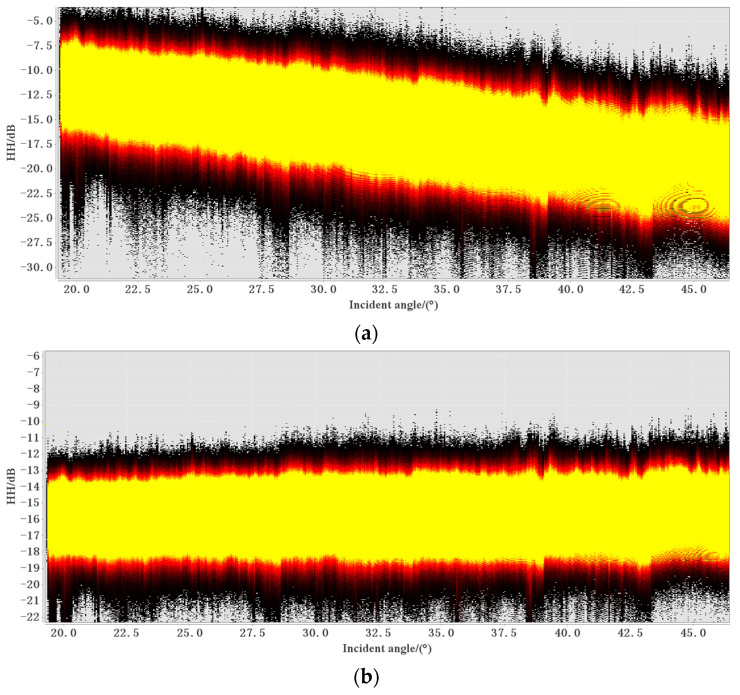
SAR image covering point A of the sonar device. (**a**) Before the normalization of the incidence angle. (**b**) After the normalization of the incidence angle.

Figure 5a shows the relationship between the backscattering coefficients and incidence angles of the pixels in Figure 4a. There are three colors in the figure, each representing the density of points. The yellow area represents the densest concentration of points, followed by the red area, and the black area has the least number of points distributed. It can be seen from the figure that there is a negative correlation between the incidence angle and the backscattering coefficient, and the backscattering value σ decreases with the increase in the incidence angle. The backscattering value σ^0^ after decibelization has a certain linear relationship with the incidence angle. The decibelization formula is shown in Equation (2) [16]:

(2) $$\sigma^{0}(\mathrm{dB})=10*\log_{10}(\sigma )$$

The incidence angle correction model is generally divided into a cosine square model and a linear model. The study area is mainly composed of seawater and sea ice, and the terrain is flat, which is suitable for the linear model [18]. The linear regression coefficients a and b were obtained according to the linear relationship between the incidence angle θ and the backscattering coefficients (σ) of Equation (3) [19]. The incidence angle of the Sentinel-1 EW data is between 19° and 47°. According to Equation (4), the symmetry value was calculated at an incidence angle of 33° (the middle value between 19 and 47). Equation (4) is a symmetric equation that describes the increase in backscattering values in the range profile in a way that is symmetrically opposite to the real decrease [19]. Finally, using the real measurement values of SAR images and the symmetric values obtained from Equation (4), all the data were theoretically normalized with reference to Equation (5) [19]. As shown in Figure 5b, after the normalization of the incidence angle, there is basically no correlation between the incidence angle and the backscattering coefficients, greatly reducing the error caused by the effect of the incidence angle.

(3) $$\sigma_{\theta }^{0}=a\theta +b$$

(4) $${\left(\sigma_{\theta }^{0}\right)}^{-1}=-a\theta +2a\theta_{ref}+b$$

(5) $$\sigma_{\theta_{ref}}^{0}=\frac{\sigma_{\theta }+{\left(\sigma_{\theta }\right)}^{-1}}{2}$$

### 3.2. Feature-Enhancement Strategy

In order to establish the relationship between the information on the SAR images and the ice thickness data, we used the following features shown in Table 2: the HH and HV features of SAR images; the HH_ref_ and HV_ref_ features of HH and HV normalized by incidence angle (as described in Section 3.1); and the ratios of HH_ref_ and HV_ref_, addition and subtraction, and normalized difference. The corresponding month, incidence angle, and sea temperature near the sonar point were used as enhancement features to predict sea ice thickness. Defining additional features helps the model to discover hidden and unknown relationships between features, facilitating thickness inversion for all ice types.

It can be seen from Section 2.2 that sea ice thickness is mainly affected by the climate and the environment of the region, changes with the change in month, and presents periodicity. Therefore, we consider sea water temperature and month to be important attributes for inverting sea ice thickness. HH polarization is more suitable for the identification of ice water than HV polarization, but in HV polarization, horizontal ice and deformed ice are easier to separate because the deformed region has a strong depolarization effect [20]. It can be seen from Section 3.1 that the incidence angle directly affects the backscattering coefficient, so HH, HV, HH_ref_, HV_ref_, and incidence angle were employed as basic features. In addition, under the influence of sea ice surface roughness, it is usually difficult to estimate ice thickness based on the backscattering coefficient [21]. Therefore, the HH_ref_ and HV_ref_ polarization ratios were used as enhancement features. The polarization ratio is related to the dielectric constant of the ice surface, a value that can reflect the difference in the dielectric constant of the ice surface caused by the change in ice thickness [11].

The reflection and scattering characteristics of sea ice with respect to the SAR signals of different polarization modes may be different. Using polarization addition and polarization subtraction, the common and difference information between different polarization modes were highlighted. The polarization normalization difference reveals the scattering intensity of one polarization mode relative to another, helping to capture the changes in scattering mechanism at the surface. The relationship between polarization characteristics and other physical properties of sea ice can be further revealed. Junhwa et al. calculated the inverse, logarithmic, Hadamard product, and normalized difference (NDTB) of brightness temperature data from different channels of AMSR2, with a total of 357 features. The daily sea ice thickness in the Arctic was inverted (with a resolution of 25 km) [3], and the results were close to the sea ice thickness values of CryoSat-2 products [22].

### 3.3. Active Learning

Due to environmental limitations, obtaining numerous sea ice thickness samples is costly, and traditional supervised learning methods often rely on a large quantity of data to effectively train models. To overcome this challenge, we adopted an active-learning [23] strategy using the maximum standard deviation sampling method. This method selects the sample with the highest standard deviation from the data pool. The higher the standard deviation, the greater the uncertainty of the result, and therefore the higher the information entropy. By incorporating these representative samples into the model for updating, the goal of attaining higher inversion accuracy with fewer samples was achieved. As shown in Equation (6), *X_i_* represents the *i*-th sample value of the day, $\bar{X}$ represents the mean value, and *SD* rsepresents the standard deviation value.

(6) $$SD=\sqrt{\frac{\sum_{i=1}^{n}{\left(X_{i}-\bar{X}\right)}^{2}}{2}}$$

**Figure 6:**
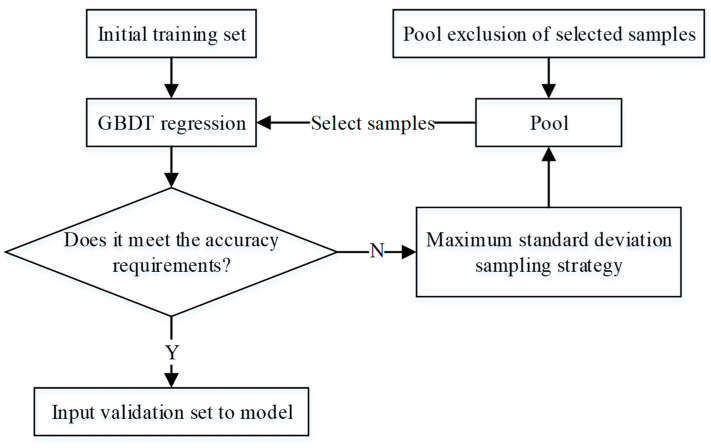
The flowchart of active learning.

As shown in Figure 6, the active learning process is as follows:

1. The sample set is initially divided into a training set and a validation set.
2. From the training set, 30% of the samples are randomly selected and used to train the GBDT model, thereby generating the initial model. The remaining 70% of the training set samples are used as a pool for sampling.
3. A sampling strategy based on the maximum standard deviation is used to select the most representative samples from the pool, they are to the model for training, and the selected samples remove are removed from the pool.
4. Check whether the training results meet the requirements. If they meet the requirements, end active learning; otherwise, skip to step 3.

Finally, input the validation set into the model to test its predictive ability.

### 3.4. IFEAL-GBDT

Choosing GBDT regression to predict sea ice thickness in the Beaufort Sea is suitable for sea ice thickness inversion tasks due to its comprehensive learning ability and ability to interpret complex features. In order to improve the performance of this model, we modified the original remote sensing data and used the modified data to generate more effective features, taking into account environmental and temporal factors. In addition, based on amplification and other information, active learning was used to select the most representative samples to input into the model for training.

**Figure 7:**
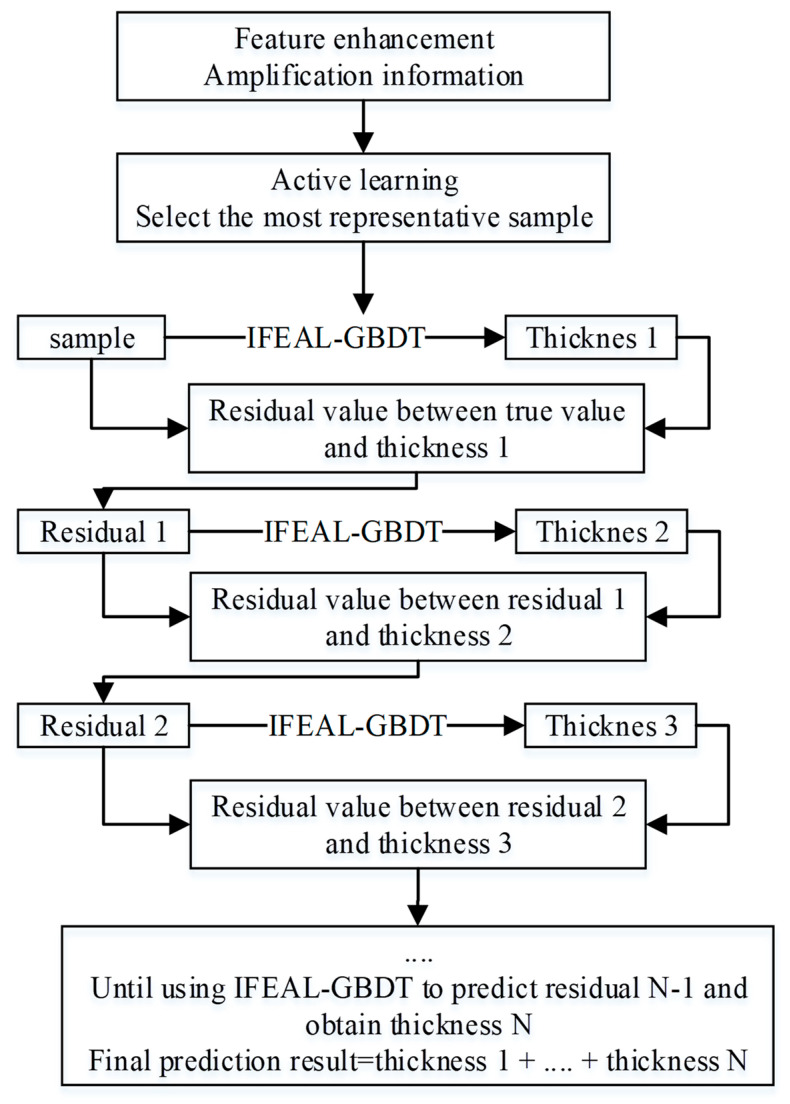
The IFEAL-GBDT prediction process.

The entire IFEAL-GBDT process is based on using feature enhancement to amplify information, then using active learning to select samples based on amplification information, and finally using the GBDT model for training and prediction. As shown in Figure 7, first, a regression tree model is randomly initialized, and the model is used to predict the training set. Based on the prediction results, the residual value of each sample is calculated (i.e., subtracting the predicted value produced by the current model from the true real value). Then, in the next step, the residual is considered the new true value, and training is performed to calculate the residual value, repeating this process. In each iteration, the tree learns to fit the missing content of the previous model, known as residuals, in order to minimize the final prediction error.

Since the inversion of sea ice thickness is usually a nonlinear problem, we can improve performance by tuning the parameters of IFEAL-GBDT to make it very robust. After many adjustments, the model parameters were set as follows: the learning rate was 0.01; as the number of iterations approached 500, the bias of the inversion results leveled off, so the number of iterations (or the number of weak learners) was set to 500; each regression estimator has a maximum depth of four; and the minimum number of samples required to split an internal node is two. The Huber loss function is a combination of the squared error for regression and the absolute error of regression.

## 4. Experimental Results and Analysis

### 4.1. Experimental Process

#### 4.1.1. Experimental Environment

The operating system of the workstation used was Windows 11. The CPU model was AMD Ryzen5 5600X, and the GPU model was NVIDIA GeForce RTX 3060. Basic preprocessing of Sentinel-1 data was performed using SNAP (Sentinel Application Platform). The Python language was used for further data processing and visualization, the Pytorch 1.10 framework and Sklearn 1.2.2 frameworks were employed using the Anaconda3 platform, and training and testing experiments were conducted using these frameworks. All the experiments were conducted in this hardware and software environment.

#### 4.1.2. Experimental Design

As mentioned in Section 2.3, there are 1854 Sentinel-1 images covering three points of sonar devices A, B, and D from 14 September 2018, to 7 September 2021, details of some SAR images are shown in Table A1 in the appendix. Since seawater may be misidentified as thin sea ice in the summer, when sea ice is very thin or even nonexistent, we removed samples with an ice thickness less than 0.02 m. In addition, the proportion of samples with an ice thickness greater than 1.8 m was very small, so we also removed these samples to improve the performance of the model. The remaining 1400 SAR images were preprocessed to generate HH and HV, and HH_ref_ and HV_ref_ were obtained via incidence angle normalization, other HH_ref_ and HV_ref_ values, the polarization ratio, the normalized difference, incidence angles, months, and seawater temperature, as described in Section 3.2.

For the sample–time matching problem, considering the large variation in ice thickness values within a day, we chose the median of all sonar measurements in a day as the label. The SAR imaging time was less than one minute. In order to reduce the influence of time, the features of all images were averaged for 10 days, and then SAR images from the same day captured with sonar devices A, B, and D were selected as samples. In Equation (7), ft represents the value of each feature on day t, and Ft represents the average value of each feature from day t − 5 to day t + 4. The dimensions and orders of magnitude of the information, month, and temperature of SAR data are different, so it is necessary to normalize the various feature values to the interval 0 to 1 to improve the reliability of each feature prediction. The normalization calculation formula is shown in (8), where F* represents the normalized value of the feature; F represents the value before normalization; and Fmin and Fmax denote the minimum and maximum values of this feature in the dataset, respectively.

(7) $$F_{t}=\frac{1}{10}\sum_{t-5}^{t+4}f_{t}$$

(8) $$F^{*}=\frac{F-F_{\min}}{F_{\max}-F_{\min}}$$

**Figure 8:**
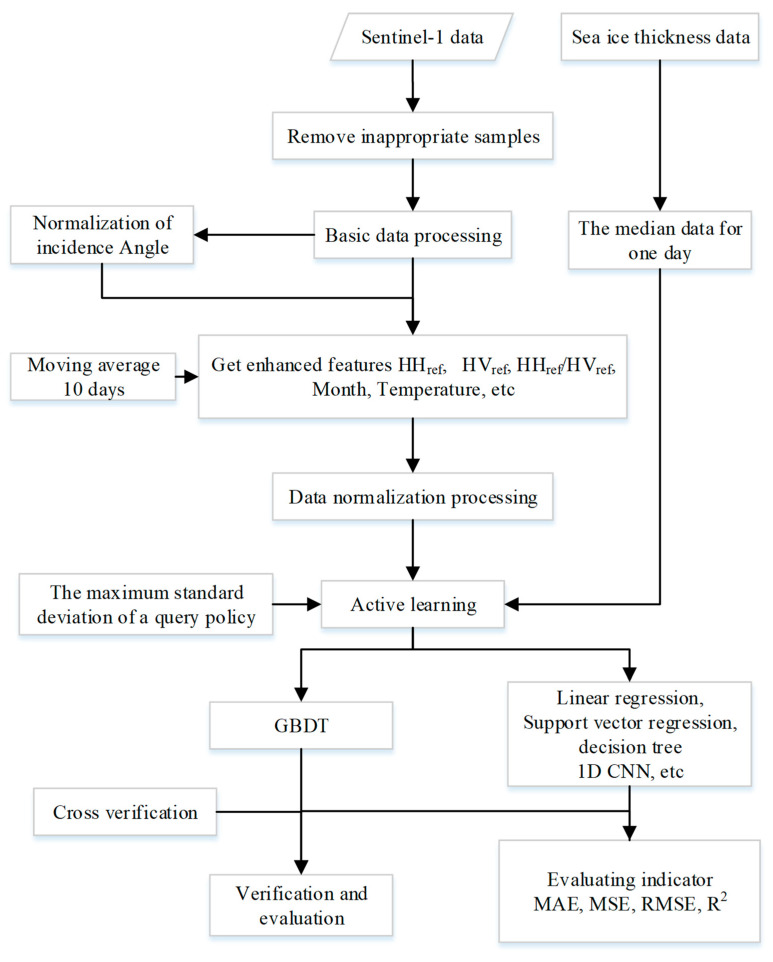
Overall experimental flowchart.

For the sample–space matching problem, we found the pixel point closest to each point of sonar devices A, B, and D from the SAR images according to the latitude and longitude and extracted the average of 3 × 3 grid pixels as the training data centered on this pixel point. Based on 3-fold cross validation, the data set was incorporated into the IFEAL-GBDT method and compared with other methods. The evaluation metrics for the final results use mean absolute error (MAE), mean square error (MSE), root mean square error (RMSE), and the coefficient of determination (R^2^). Smaller MAE, MSE, and RMSE values indicate a better prediction effect of the regression model. R^2^ is used to judge the fitting effect of the regression model. The closer it is to 1, the better the fitting effect of the model. The overall experimental process described above is shown in Figure 8.

### 4.2. Comparative Experimental Analysis

In order to verify the effectiveness and advantages of the proposed method in relation to sea ice thickness inversion, we used the same data set to conduct comparative experiments on related methods. The comparison methods included linear regression (LR), Bayesian linear regression (BR), Support Vector Regression (SVR), decision trees (DTs), a Back Propagation Neural Network (BPNN), and a 1D Convolutional Neural Network (1DCNN); these methods also use feature enhancement and active learning. The parameters of the models are as follows: the alpha value of the BR model is 1, and the lambda value is 0.001. The SVR model uses a Gaussian kernel function. The loss function of the DT model is the mean absolute error regression loss, and the maximum depth of the tree is 4. The activation function of the BPNN model is a rectified linear unit function, the weight update optimizer is Adam (a method for stochastic optimization), and the learning rate is 0.005. The structure of a 1D Convolutional Neural Network consists of three layers, each containing one 1D convolution, one 1D pooling, and one rectified linear unit activation function.

As described in Section 4.1.2, we shuffled the dataset data and adopted three-fold cross validation, where the quantities of training set and test set samples were 933 and 467, respectively. The experimental results for the different methods are shown in Table 3. It can be seen from the table that the traditional mathematical models, such as LR and BR, yield average performance in sea ice thickness inversion, with RMSEs of 24.3 and 22.6 cm, while the root mean square error of the DT, BPNN, and SVR models decreases from 17 cm to 16 cm, and the R^2^ increases from 0.84 to 0.86. The correlation between SAR image features and sea ice thickness can be basically fitted. The DT, BPNN, and SVR models exhibit great improvement compared with the traditional mathematical models, proving that the machine learning model offers significant advantages in terms of the inversion of sea ice thickness. It is worth noting that the results for the deep learning model 1DCNN are only better than those for the linear model because the advantages of deep learning are not obvious when the samples are small. The IFEAL-GBDT method exhibited the best performance, with an MAE, an MSE, and an RMSE as low as 8.5 cm, 1.9 cm, and 13.7 cm, respectively, and an R^2^ as high as 0.912.

**Figure 9:**
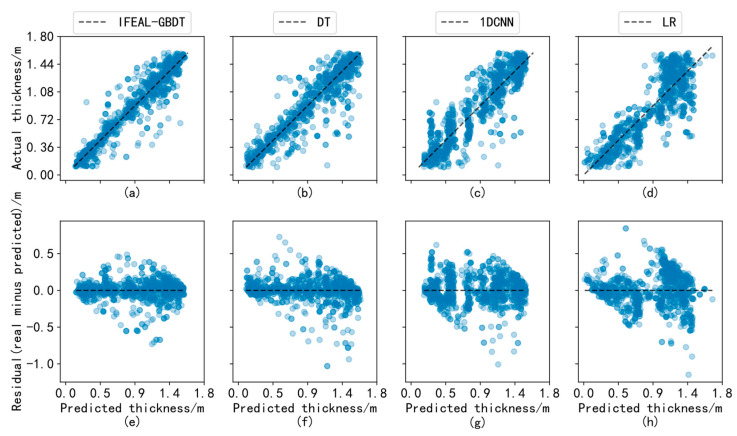
Analysis of the experimental results for IFEAL-GBDT, DT, 1DCNN, and LR. (**a**) Scatter plot of predicted and true values of IFEAL-GBDT; (**b**) scatter plot of predicted and true values of DT; (**c**) Scatter plot of predicted and true values of 1DCNN; (**d**) Scatter plot of predicted and true values of LR; (**e**) Scatter plot of predicted and residual values of IFEAL-GBDT; (**f**) Scatter plot of predicted and residual values of DT; (**g**) Scatter plot of predicted and residual values of 1DCNN; (**h**) Scatter plot of predicted and residual values of LR.

The examples in Figure 9 show analysis diagrams of the experimental results for the IFEAL-GBDT method, DT, 1DCNN, and linear model (due to limited space, four methods with relatively large differences in their results are shown). The first row is a scatter plot of the predicted and true values for each method. The second row is a scatter plot of the differences between the predicted and true values for each method. The *X*-axis represents the predicted value of the experiment, the *Y*-axis in the first row represents the true value of the sample, and the *Y*-axis in the second row represents the difference between the true value of the sample and the predicted value. The closer the predicted value of the sample is to the black line (the best fit line), the smaller the deviation from the true value. It can be seen from the figure that the maximum sea ice thickness of 1.6 m can be inverted. Most of the points fall near the best fitting line, and the overall deviation is minimal, proving the superiority of the IFEAL-GBDT method in sea ice thickness inversion. The second-best performance was exhibited by the decision tree model, and the linear model’s performance was average.

### 4.3. Feature Importance Analysis

In this experiment, sea ice thickness was inverted using multiple features, but it is difficult to physically quantify the importance of individual features. Even if a feature is of low importance, it may have hidden and unknown relationships with other features. To this end, we conducted a feature importance analysis to study the correlations between the features and between the features and sea ice thickness. We used the Pearson coefficient to calculate the correlation between the individual features, and the correlation coefficient ranges from −1 to 1. A negative value indicates that as the value of one variable increases, the other decreases, showing a negative correlation; a positive value means that as the value of one variable increases, the other one also increases, and this relationship is positively correlated. A value of 0 means that a change in one feature has no effect on the value of the other. The Pearson coefficient is shown in Equation (9), which calculates the quotient between the covariance and standard deviation of the sample, where feature $\bar{X}$ is the average of n features *X_i_*, feature $\bar{Y}$ is the average of n features *Y_i_*, and *r* is the Pearson coefficient between the two features.

(9) $$r=\frac{\sum_{i=1}^{n}\left(X_{i}-\bar{X})(Y_{i}-\bar{Y}\right)}{\sqrt{\sum_{i=1}^{n}{\left(X_{i}-\bar{X}\right)}^{2}}\sqrt{\sum_{i=1}^{n}{\left(Y_{i}-\bar{Y}\right)}^{2}}}$$

**Figure 10:**
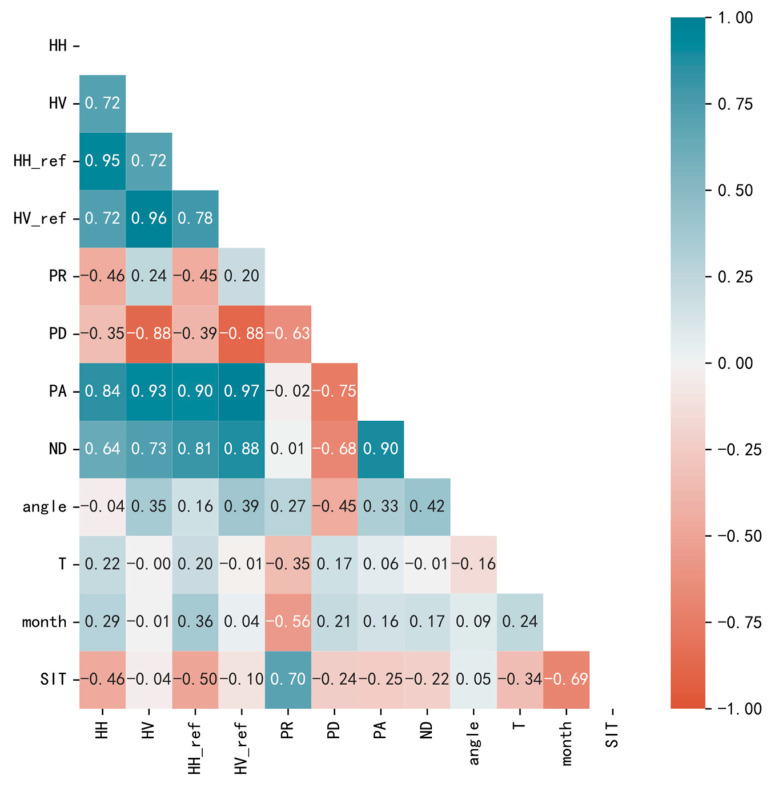
Heat map between features. Black numbers indicate relatively low correlations, and white numbers indicate relatively high correlations.

The degree of correlation is divided into five degrees: 0.8–1 is a strong correlation; 0.6–0.8 is also a strong correlation; 0.4–0.6 is a moderate correlation; 0.2–0.4 is a weak correlation; and 0–0.2 is a very weak correlation or no correlation. Figure 10 shows heat maps between 11 features and sea ice thickness (SIT) based on the Pearson coefficient.

Here, PR is the polarization ratio, PD is the polarization difference, PA is the polarization addition, ND is the polarization normalization difference, T is the seawater temperature, and angle is the local incidence angle. The lighter the color, the lower the correlation, with blue representing a positive correlation and red representing a negative correlation. Among them, PR and month have the best correlation with thickness value, and the correlation coefficient is 0.7, which is a strong correlation. HH and HH_ref_ are generally negatively correlated with thickness values, and the corresponding correlation coefficients are −0.46 and −0.5, respectively, indicating a moderate correlation. It can be seen that there is an influence of the incidence angle, and the HH_ref_ has a higher correlation than the unnormalized HH. For the variation in the surface roughness of ground objects, co-polarized HH is more sensitive than cross-polarized HV [24], and cross-polarized HV images have strong noise [25]. PD, PA, ND, T (with a weak correlation), HV, HV_ref_, angle (with a very weak correlation), and SIT have low correlations, but there is a strong correlation between each feature. Although HV and HV_ref_ have low correlation with SIT, they have high correlations with HH, HH_ref_, PD, PA, and ND, reaching the degree of a strong correlation. The local incidence angle and PD, ND, month, and PR are all moderately correlated.

A T-test, which is a hypothesis-testing method commonly used in statistics to determine whether observed data differences are caused by random errors or indeed reflect substantive relationships between variables, was used to test the significance of each feature. For each feature, we calculated the *p*-value (denoting statistical significance). The *p*-value of the incidence angle feature is 0.0577, and the incidence angle itself acts as an auxiliary feature, which can provide supplementary information when other features are affected. The rest of the features have *p*-values well below 0.0001, which is considered significant (assuming there is a significance level of 0.05).

**Figure 11:**
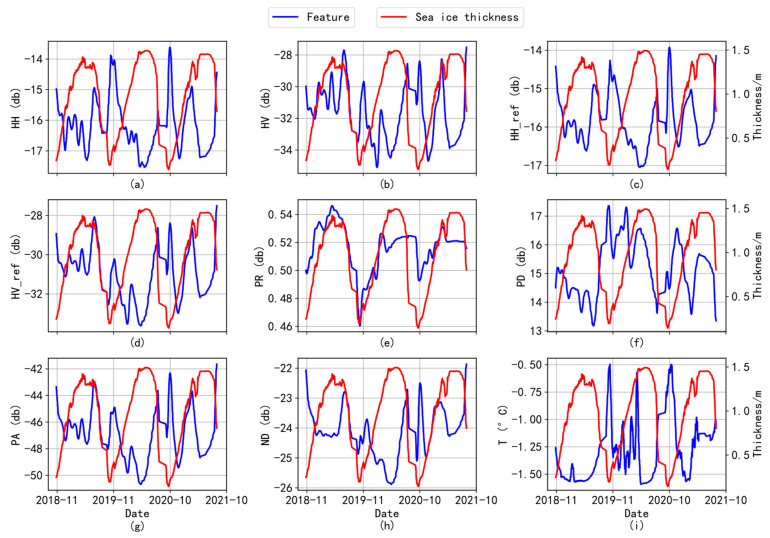
The trend relationship between different characteristics and sea ice thickness. (**a**) Curve of HH varying with sea ice thickness; (**b**) curve of HV varying with sea ice thickness; (**c**) curve of HH_ref_ varying with sea ice thickness; (**d**) curve of HV_ref_ varying with sea ice thickness; (**e**) curve of PR varying with sea ice thickness; (**f**) curve of PD varying with sea ice thickness; (**g**) curve of PA varying with sea ice thickness; (**h**) curve of ND varying with sea ice thickness; (**i**) curve of T varying with sea ice thickness.

Since some samples may have local incidence angles around 33°, HH and HV do not need to be normalized, and normalization will introduce errors, so the unnormalized features (HH and HV) were added. In Figure 11, the *X*-axis is the date, the left *Y*-axis is the characteristic value, and the right *Y*-axis is the sea ice thickness value. The blue line represents the change in the characteristic value, and the red line represents the change in thickness value. It can be seen from Figure 11a,b that the characteristic values for HH and HV also peak when the sea ice thickness reaches the trough interval. This is because radar signals from HH and HV polarization patterns can penetrate sea ice better when it is thinner. HH polarization constitutes horizontal emission and reception, both being parallel to the sea ice surface and more susceptible to surface roughness and therefore more sensitive to sea ice thickness. The cross-polarized HV is less affected by the sea state and has certain advantages in distinguishing the boundary of seawater and sea ice (with a smaller thickness value).

When the ice thickness value increases, the backscattering coefficient will be affected by many aspects (sea ice salt concentration, surface roughness, time, and incidence angle). It can be seen from Figure 11c,d that HH and HV are more sensitive to changes in thick ice after the normalization of incidence angles. The change in the PR trend in Figure 11e is similar to the change in SIT. When the salt content of sea ice decreases with the increase in thickness value, the decrease in the dielectric constant makes the polarization ratio very sensitive [11]. Figure 11f has a nonlinear relationship with sea ice thickness, with obvious fluctuations. The trends of feature change in Figure 11d,g are almost the same, but the ranges of feature values are different. HH_ref_ ranges from −34 dB to −28 dB, and PA ranges from −51 dB to −42 dB. When sea ice melts, the variation trend of the eigenvalues in Figure 11h is opposite to the trend of sea ice thickness, the curve of eigenvalues in the interval is smooth, and the zigzag degree is small. Figure 11i clearly shows that the sea ice begins to melt when the sea water heats up, and sea ice begins to form when the sea water cools down. Therefore, adding different characteristic parameters to improve the resolution of e sea ice type is very important for sea ice thickness inversion.

### 4.4. Analysis of Different Feature Combination Results

The distribution of sea ice thickness is affected by many factors, such as sea state, so its thickness distribution is complicated. However, the backscattering characteristics of sea ice usually have high consistency and low differentiation, and it is often impossible to obtain satisfactory results by relying only on a single attribute. Different feature parameters contain different information on ground objects. Therefore, combining these feature parameters to construct feature vectors, giving full play to their respective feature advantages and allowing them to complement each other, is conducive to improving the regression effect. To this end, a feature-enhancement strategy is proposed in this paper, and it was discussed in detail in Section 3.2. In order to verify the correctness of the feature-enhancement strategy proposed in this paper with respect to sea ice thickness inversion, four combination schemes are considered: (1) using HH and HV without normalization of incidence angles; (2) using the normalized HH_ref_ and HV_ref_ of incidence angles and the extended four features; (3) using a combination of F1 and F2; and (4) using all features. The experimental schemes are shown in Table 4.

The experimental results for the different methods under different feature combinations are shown in Table 5 (the case of feature combination F4 was reported in Section 4.2). In the case of feature combination F1, the linear regression model and the Bayesian classification model performed the worst, with an RMSE and an R^2^ of around 0.344 cm and 0.40, respectively. Limited by the kernel function, the prediction performance of the SVR model is not excellent, with an RMSE and an R^2^ of 0.317 cm and 0.489, respectively. When the amount of feature information provided is very small, the 1DCNN model has difficulty extracting effective information in the case of complex structures, and the RMSE and R^2^ are 0.337 cm and 0.425, respectively. Similarly, the decision tree model is not ideal for separating signals and noise with less information, with an RMSE and an R^2^ of 0.334 cm and 0.435, respectively. The BPNN and GBDT models performed well, with RMSEs of 0.297 cm and 0.277 cm and R^2^ values of 0.552 and 0.61, respectively. HH_ref_ and HV_ref_ are the theoretical backscatter values unified under the incidence angle of 33°, which reduced the influence of different incidence angles on the backscatter values and generated smaller errors. Therefore, the overall effect of feature combination F2 is significantly greater than that of feature combination F1. F2 uses more enhanced features and contains more information, and the overall RMSE decreases by 7–10 cm, and DT decreases by about 13 cm, with an RMSE and an R^2^ of 0.206 cm and 0.785, respectively. The GBDT model is still the best, with an RMSE and an R^2^ of 0.177 cm and 0.84, respectively.

Feature combination F3 further improves ice water recognition ability by constructing a feature vector via the fusion of the features of F1 and F2. F3’s role was mainly to reduce the abnormal predicted value and the error caused by the incidence angle normalization of a small number of samples that were originally around 33°. Compared with other methods, GBDT still has the best effect, with an RMSE and an R^2^ of 0.176 cm and 0.842, respectively. Comparing the results for feature combinations F1, F2, and F3 and adding feature combination F4 obtained after attaining the incidence angle, temperature, and time, IFEAL-GBDT achieves the best effect in terms of error size and correlation.

### 4.5. Analysis of Active-Learning and Extrapolation Experiments

To evaluate the effect of active learning on the generalization ability of the model, as shown in Table 6, the dataset is not shuffled, unlike that described in Section 4.2. As shown in Table 6, the samples from October 2018 to August 2020 were used as the training set (889), and the samples from September 2020 to September 2021 were used as the prediction set (511). Both (1) and (2) use feature enhancement; (1) is the result without active learning, and (2) is the result with active learning.

The analysis in Table 6 shows that the performance ability of each model was significantly improved after screening representative samples through active learning. In the extrapolation experiment, because the samples of the first 23 months were used to predict the samples of the next 12 months, the time span is large, and the number of training sets is small, active learning can make up for this disadvantage. For the BPNN and DT models, the R^2^ is higher than 0.7, and the RMSE is between 21 cm and 24 cm. Although the results for the other methods improved after the use of active learning, the overall effect is still average. IFEAL-GBDT, proposed in this paper, still yielded the best results, with an R^2^ reaching 0.836 and an RMSE of 17.6 cm. Compared with the strategy without active learning, the R^2^ is 0.782, and the RMSE is 20.2 cm, and the improvement is still obvious.

**Figure 12:**
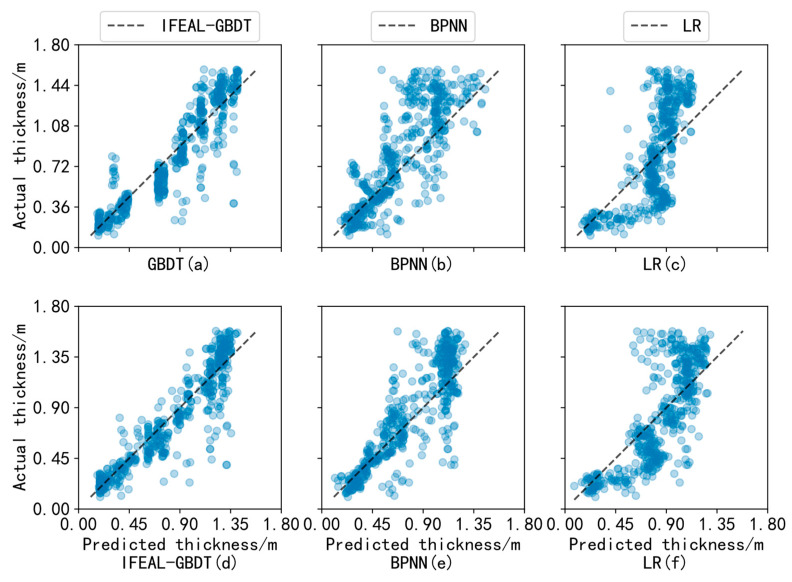
Analysis of experimental results for active learning in extrapolation experiments.

As shown in Figure 12, in the extrapolation experiment, the resulting plots for the GBDT, BPNN, and LR models that were not paired with active learning are shown in Figure 12a, Figure 12b, and Figure 12c, respectively, and those with active learning are shown in Figure 12d, Figure 12e, and Figure 12f, respectively. Some samples contain highly similar feature information, which leads to a redundancy of feature variables and reduced separation of sea ice type and thickness, so the prediction results are clustered together. In Figure 12a, this phenomenon exists between 36 cm and 72 cm on the *Y*-axis, where the blue points are concentrated in one block. Active learning is used to select the samples with the largest standard deviation, and the samples with larger standard deviations have higher uncertainty and contain higher information entropy, as shown in Figure 12d, reducing the occurrence of this phenomenon, and the same is true for Figure 12e,f.

When the value is below 1.2 m, the predicted value is larger than the actual value; this is due to climatic reasons. For example, during the summer ice melt, it is easy to identify the sea water at the ice–water intersection as sea ice [26]. In addition, the weak reflectivity of thin ice leads to the absorption of SAR signals when passing through thin ice, so the scattering value will be small. However, as can be seen from Figure 11, the feature is basically negatively correlated with thickness, so the predicted value will be too large. Similarly, above 1.2 m, the reflection ability of thick ice is strong, and the scattering value will be large, so the predicted value will be small. Lower predicted values than the actual values were mainly obtained during the thick-ice period (February to July).

## 5. Conclusions

SAR has all-day and all-weather capabilities, and radar waves can penetrate clouds and fog and are not affected by weather, providing a good technical means for large-scale sea ice detection. However, due to the limitations of environmental conditions in the actual sea ice distribution area, it is extremely difficult to obtain large-scale and long-term measured data. Moreover, the band of SAR image data is relatively single, and the information contained in them is limited. If the remote sensing data themselves contain noise, errors, or uncertainty, the inversion results will also be affected. In order to fully exploit the features of sea ice SAR remote sensing images and further improve the accuracy of sea ice thickness retrieval, we propose an inversion method based on active learning and a feature-enhancement strategy combined with GBDT, providing a new idea for the application of remote sensing to sea ice thickness retrieval. The experimental results show that the proposed IFEAL-GBDT method can make full use of the information on different features with fewer training samples and obtain the best inversion accuracy in general, compared with most inversion methods using a single attribute and other sea ice thickness retrieval methods.

(1) The information provided by remote sensing data is limited, only consisting of dual-polarization features HH and HV, and it is easily affected by the incidence angle. Therefore, the incidence angle was used to correct the original polarization information from space. Considering that this information constitutes long time-series data with a large time span, the centered moving average for all features was 10 days, and the window size was also 10 days. Sea ice thickness varies greatly within a day, and the SAR imaging time does not coincide with the actual measurement time of sea ice thickness, so the median of sea ice thickness in a day was chosen to represent the actual thickness of that day. Multi-attribute features, such as the polarization ratio, polarization normalized difference, and polarization difference, were generated based on the correction information. Considering the influence of the climate of an environment, month and sea temperature attributes were added to increase the separability of sea ice with different thicknesses.

(2) Through the feature-enhancement strategy, the feature information of the sample was increased, compensating for the limitations of using the backscatter coefficient to retrieve sea ice thickness. Then, the active-learning strategy based on maximum standard deviation was studied. According to the correction and increased information, the most valuable samples were selected for training, addressing the limiting effect of a small sample size on the improvement in thickness inversion accuracy. In the face of a complex geographical environment and the physical properties of sea ice, the advantages of the GBDT model for dealing with nonlinearity, high dimensionality, and data noise were used to reduce the bias of a single model. Active learning and feature enhancement have inherent consistency in improving regression accuracy, on the one hand increasing the original effective information and, on the other hand, sampling the most informative samples; the combination of these advantages with GBDT allows the model to provide optimal performance.

Further research is needed to consider the physical characteristics and formation mechanisms of sea ice. For example, dark nilas is affected by wind and waves, easily broken, and dark in color. Grey ice is less elastic than newborn ice and breaks when it expands. Grey ice is more likely to form ridges under pressure, which will affect the reflection and scattering of remote sensing signals. Other factors, such as sea ice melting in the summer and snow-covered sea ice in the winter, also affect the retrieval of sea ice thickness.

## Figures and Tables

**Table 1:** Specific information on the longitude, latitude, and times of the three ULS points.

Device	A	B	D
coordinate	75.0001° N 149.9994° W	78.0054° N 149.9641° W	74.0031° N 140.0019° W
time frame	25 September 2018–27 August 2021	23 September 2018–30 August 2021	14 September 2018–7 September 2021
duration	1074	1073	1089

(A: Sonar device A, B: Sonar device B, D: Sonar device D).

**Table 2:** Features and descriptions used in sea ice thickness inversion models.

Id	Feature	Description
1	HH, HV	Dual polarization characteristics of Sentinel-1
2	HH_ref_, HV_ref_	Normalization of HH and HV by incidence angle
3	HH_ref_ − HV_ref_	Polarization subtraction
4	HH_ref_ + HV_ref_	Polarization addition
5	HH_ref_/HV_ref_	Polarization ratio
6	Id 3/Id 4	Normalized difference
7	Incidence angle	Corresponding incidence angle
8	Month	Corresponding month
9	Temperature	Sea water temperature

(Id3/Id 4: Feature Id 3 divided by feature Id 4).

**Table 3:** Comparison of results of various methods in validation experiments.

Method	MAE	MSE	RMSE	R^2^
LR	0.188	0.059	0.243	0.702
BR	0.167	0.05	0.226	0.732
1DCNN	0.151	0.04	0.201	0.793
SVR	0.114	0.029	0.172	0.845
BPNN	0.112	0.027	0.165	0.861
DT	0.102	0.026	0.163	0.866
IFEAL-GBDT	0.085	0.019	0.137	0.912

(MAE: mean absolute error, MSE: mean square error, RMSE: root mean square error, and R^2^: coefficient of determination).

**Table 4:** Four feature combinations.

Feature Vector	Expression
F1	HH, HV
F2	HH_ref_, HV_ref_, HH_ref_ ± HV_ref_, HH_ref_/HV_ref_, (HH_ref_ − HV_ref_)/(HH_ref_ + HV_ref_)
F3	F1 + F2
F4	F3, Incidence angle, Month, Temperature

(F1 + F2: the features of F3, including F1 and F2).

**Table 5:** Results of different methods under different feature combinations.

Methods	F1		F2		F3		F4	
	RMSE	R^2^	RMSE	R^2^	RMSE	R^2^	RMSE	R^2^
LR	0.344	0.401	0.263	0.649	0.261	0.654	0.243	0.702
BR	0.344	0.403	0.264	0.646	0.263	0.65	0.226	0.732
1DCNN	0.337	0.425	0.239	0.706	0.237	0.714	0.201	0.793
SVR	0.317	0.489	0.215	0.766	0.212	0.771	0.172	0.845
BPNN	0.297	0.552	0.194	0.809	0.192	0.813	0.165	0.861
DT	0.334	0.435	0.206	0.785	0.217	0.761	0.163	0.866
IFEAL-GBDT	0.277	0.61	0.177	0.84	0.176	0.842	0.137	0.912

(RMSE: root mean square error; R^2^: coefficient of determination).

**Table 6:** Model use and lack thereof with active learning in generalization experiments.

Methods	(1)		(2)	
	RMSE	R^2^	RMSE	R^2^
LR	0.322	0.456	0.287	0.564
BR	0.323	0.453	0.313	0.484
1DCNN	0.286	0.571	0.246	0.686
SVR	0.292	0.551	0.248	0.676
BPNN	0.285	0.573	0.235	0.709
DT	0.268	0.622	0.218	0.754
IFEAL-GBDT	0.202	0.782	0.176	0.836

((1): Using active learning, (2): active learning was not used).

## Data Availability

Data are contained within the article.

## Appendix A

The detailed information on the Sentinel-1 data used in this article is shown in Table A1, including file ID, Date, Path, and Frame.

**Table A1:** Details of the Sentinel-1 data.

File ID	Date	Path	Frame
S1A_EW_GRDM_1SDH_20181214T164117_20181214T164217_025024_02C28F_7842-GRD_MD	14 December 2018	102	334
S1A_EW_GRDM_1SDH_20190109T162547_20190109T162641_025403_02D04E_65E7-GRD_MD	9 January 2019	131	346
S1B_EW_GRDM_1SDH_20190417T165724_20190417T165824_015849_01DC42_4BEB-GRD_MD	17 April 2019	73	338
S1B_EW_GRDM_1SDH_20201107T174548_20201107T174648_024162_02DEE4_E72F-GRD_MD	7 November 2020	161	324
S1A_EW_GRDM_1SDH_20201202T173801_20201202T173905_035510_0426DA_FA73-GRD_MD	2 December 20201	88	321
S1A_EW_GRDM_1SDH_20210103T163414_20210103T163514_035976_0436F8_7AA9-GRD_MD	3 January 2021	29	346
S1B_EW_GRDM_1SDH_20210312T175351_20210312T175458_025985_031991_A085-GRD_MD	12 March 2021	59	322
